# IgE-Mediated Reaction to Levamisole: Evaluation of a Patient With Severe Anaphylaxis

**DOI:** 10.7759/cureus.17815

**Published:** 2021-09-08

**Authors:** Wendy T Garzon-Siatoya, Ismael Carrillo-Martin, Mario Rodenas, Alexei Gonzalez-Estrada

**Affiliations:** 1 Division of Pulmonary, Allergy, and Sleep Medicine, Department of Medicine, Mayo Clinic, Jacksonville, USA; 2 Division of Rheumatology, Department of Medicine, University of Florida, Gainesville, USA

**Keywords:** anaphylaxis, angioedema, adjuvant therapy, hypersensitivity reactions, skin tests, desensitization

## Abstract

Levamisole has been used as adjuvant immunomodulatory therapy for certain conditions such as amyotrophic lateral sclerosis (ALS). We present a case of a 70-year-old man with ALS who was started on levamisole with adequate response. Within 10 days of treatment, he developed a maculopapular non-pruritic rash on his extremities, and the medication was discontinued. However, two days later, he developed angioedema of the face and hands, urticaria in the extremities and torso, and throat closing sensation that was successfully treated in the emergency department with epinephrine, systemic corticosteroids, and antihistamines. Eight hours later, he presented with recurrent facial angioedema. He was transferred to the ICU and received two more doses of epinephrine and intravenous methylprednisolone. The patient fully recovered within 72 hours and was discharged with the indication to avoid levamisole. One month after the reaction, skin tests (prick and intradermal) with 10-fold dilutions of 550 mg/mL levamisole were positive at a concentration of 55 mg/mL (1:10 dilution). Since the patient developed anaphylaxis and tested positive for levamisole on intradermal testing, and after discussing the options with him, we decided to advise against using this medication since the benefits did not outweigh the risks of administration. This case highlights that IgE-mediated reactions to levamisole, while rare, can occur and be life-threatening. Shared decision-making should be done between patients and physicians after open, evidence-based discussions.

## Introduction

Amyotrophic lateral sclerosis (ALS) is a neurodegenerative disease characterized by progressive muscular paralysis reflecting degeneration of motor neurons in the primary motor cortex, corticospinal tracts, brainstem, and spinal cord [1]. Levamisole, an anthelmintic agent with a wide range of immunomodulatory properties, has been used off-label as monotherapy and an adjunct to treatment in various diseases, including ALS [2]. We present a case of severe anaphylaxis secondary to levamisole with a positive skin test. The specific diagnostic approach is examined, including non-irritating concentrations for levamisole.

This article was previously presented as a meeting abstract at the American College of Allergy, Asthma, and Immunology 2020 Annual Scientific Meeting on November 13-15, 2020.

## Case presentation

**Figure 1:**
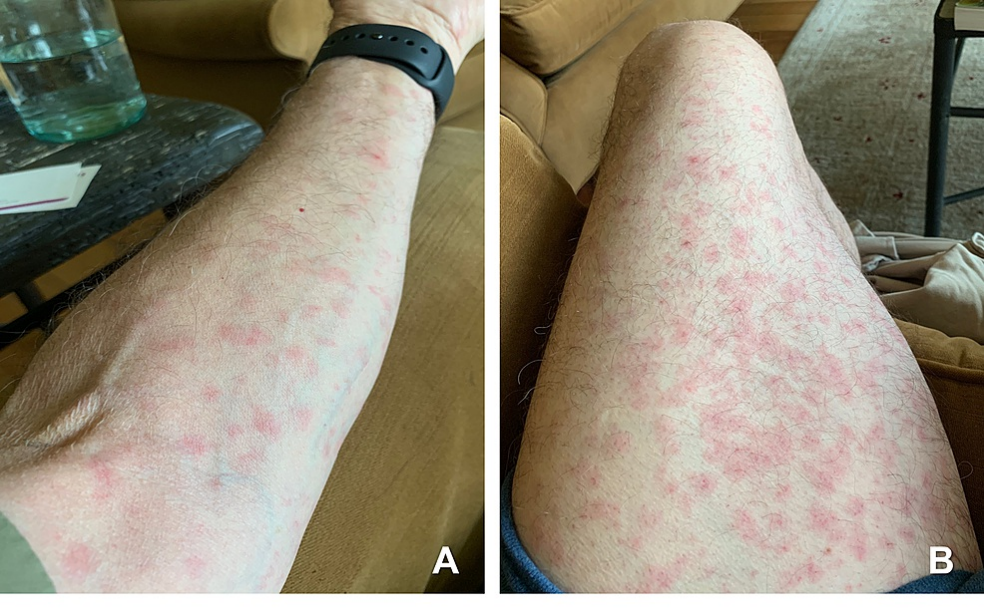
Initial presentation of the reaction (A and B) Maculopapular non-pruritic rash on the patient’s extremities.

**Figure 2:**
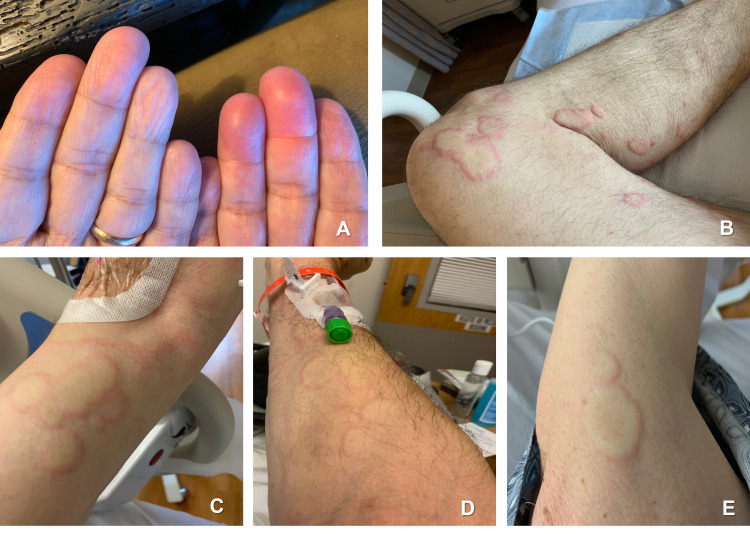
Evolution of the allergic reaction 48 hours later (A) Digital angioedema, (B-E) urticaria of upper and lower extremities.

A 70-year-old man with ALS was prescribed adjuvant levamisole (137.5 mg daily) at an outside medical institution with adequate symptomatic control. Within 10 days of treatment, he developed a maculopapular non-pruritic rash on his extremities, and the medication was discontinued (Figures 1A, 1B). However, two days later, he developed facial and digital angioedema (Figure 2A), urticaria of chest, upper and lower extremities (Figures 2B-2E), and throat closing sensation. He required an emergency department visit where he was treated with intramuscular (IM) epinephrine, intravenous (IV) dexamethasone, and diphenhydramine with adequate symptom improvement.

Approximately eight hours later, he developed recurrent facial angioedema involving lips and tongue with associated cough. The patient was transferred to the intensive care unit, where he received two more doses of epinephrine as well as IV methylprednisolone. There was no need for intubation, and the patient fully recovered over the course of 72 hours. The patient was discharged with the recommendation to avoid levamisole and referred to the outpatient allergy and immunology clinic. A tryptase level was obtained during the acute event, which was elevated 12.7 ug/L (reference value ≤ 10.9 ug/L). There was no baseline tryptase as reference.

The patient has no history of asthma, eczema, chronic urticaria, allergies to food, or medications. During the reaction to levamisole, the laboratory workup for hereditary angioedema was negative (normal levels of complement C4, C1q, C1 inhibitor level, and function). The possibility of an allergic reaction to other medications was also evaluated. An in-depth review of the medications taken by the patient before the index reaction was made. The patient was taking vitamin B12, magnesium, and Basis (a telomere extending supplement) for over a year without complications. The patient denied using NSAIDs, ACE inhibitors, or beta-blockers around the time of the event.

The patient was evaluated at our allergy clinic one month after the reaction, and we performed skin tests (prick and intradermal). The tests were performed on the volar forearm skin of the patient as indicated in current international guidelines [3]; for the prick test, we used an undiluted 550 mg/ml (stock) concentration of levamisole. The stock concentration was prepared by disintegrating the tablet in a mortar. The intradermal test was performed with 10-fold dilutions (1:1000, 1:100, and 1:10) of the stock diluted in 0.9% normal saline, then filtered using Millex-GV, Durapore® hydrophilic polyvinylidene fluoride (PVDF) membrane (0.22 µm) (Merck Millipore Ltd. Tullagreen, Carrigtwohill, County Cork, Ireland). The patient tested positive at an intradermal concentration of 55 mg/mL (1:10 dilution) after 15 minutes (Table 1). The skin test was considered positive after an increase in diameter of the initial wheal by 3 mm or greater compared to the control with an associated flare [4].

**Table 1:** Skin testing protocol for levamisole SP: skin prick; ID: intradermal. *Undiluted (stock) concentration. **10-fold dilutions from stock levamisole were made with 0.9% normal saline.

Test	Concentration	Result
SP	550 mg/mL^*^	Negative
ID	0.55 mg/mL^**^	Negative
ID	5.5 mg/mL^**^	Negative
ID	55 mg/mL^**^	Positive

The 55 mg/mL intradermal concentration was negative in six healthy volunteers. Consequently, he was diagnosed with a levamisole-induced IgE-mediated hypersensitivity reaction.

The patient was invited to participate in the medical decision process. After a thorough discussion, we concluded that the benefits of using this medication did not outweigh the risks of performing an oral provocation test or induction of tolerance to levamisole. He decided to avoid the medication and use equally efficacious alternatives.

## Discussion

There are no standardized protocols or substantive data about the optimal drug concentration available for skin testing using a drug solution prepared from an oral formulation [4]. Penicillin and a limited number of other medications (e.g., insulin) are the only agents with established optimal non-irritant drug concentrations [5]. The non-irritant drug concentration should ideally be established in healthy controls [4]. In this case, with the information gathered from the results of the skin tests performed on the healthy subjects, we can infer that the patient’s skin test findings were not the result of an irritant effect of levamisole.

Levamisole is thought to exert its effects on the immune system through cholinergic activity on T lymphocytes increasing lymphocyte cyclic guanosine monophosphate (cGMP). An increase in adenosine deaminase and a "scavenger" effect on free radicals are also felt to play a role in the actions of levamisole. The drug has a wide range of immunomodulatory applications resulting from its ability to stimulate and suppress immune responses to different antigens and its actions on a variety of immune cell types [6].

Allergic reactions to levamisole have seldom been reported in the literature. Skin rashes and dermatitis are listed as adverse reactions in reports on levamisole toxicity [7,8]. Other common side effects include gastrointestinal symptoms such as nausea and abdominal pain, flu-like syndrome, and arthralgias [7]. Levamisole has also the potential of agranulocytosis, multifocal leukoencephalopathy, ataxia, psychosis, myopathy, lichenoid eruptions, leg ulcers, fixed drug eruptions, necrotizing vasculitis, and retiform purpura [7,9,10].

The occurrence of symptoms consistent with IgE-mediated reactions to levamisole may warrant evaluation in cases where the patients are likely to continue requiring the medication by induction of drug tolerance (desensitization). Patients should be included in the medical decision process after open, evidence-based discussions.

## Conclusions

Levamisole is an agent with immune-boosting function potential that has yet to find a firm place in the treatment of ALS and remains a possible culprit of severe anaphylactic reactions. In cases such as our patient’s, the more sensitive drug provocation test could work as diagnostic confirmation. However, the performance of this test should always consider whether or not its result will provide more valuable information than it presents risks.

Consistently, desensitization protocols allow some patients to continue using medications to which they are allergic. Performing these also presents with risks that in patients with severe reactions or where the drug is not the first line of treatment might not be outweighed by the benefits of continuing to use the medication.

An optimal nonirritant drug concentration and negative predictive value for IgE-mediated reactions to levamisole have not been established and should be further determined in healthy controls.
